# Vitamin A Deficiency Impairs Mucin Expression and Suppresses the Mucosal Immune Function of the Respiratory Tract in Chicks

**DOI:** 10.1371/journal.pone.0139131

**Published:** 2015-09-30

**Authors:** Xiaoxiao Fan, Shaoqiong Liu, Guanhua Liu, Jingpeng Zhao, Hongchao Jiao, Xiaojuan Wang, Zhigang Song, Hai Lin

**Affiliations:** Department of Animal Science, Shandong Agricultural University, Shandong Key Lab for Animal Biotechnology and Disease Control, Taian, Shandong, 271018, China; Indian Institute of Science, INDIA

## Abstract

The chicken immune system is immature at the time of hatching. The development of the respiratory immune system after hatching is vital to young chicks. The aim of this study was to investigate the effect of dietary vitamin A supplement levels on respiratory mucin and IgA production in chicks. In this study, 120 one-day-old broiler chicks were randomly divided into 4 groups consisting of three replicates of 10 broilers and subjected to dietary vitamin A supplement levels of 0, 1,500, 6,000, or 12,000 IU/kg for seven days. Compared with control birds, vitamin A supplementation significantly increased the mucin and IgA levels in the bronchoalveolar lavage fluid (BALF) as well as the IgA level in serum. In the lungs, vitamin A supplementation downregulated TNF-α and EGFR mRNA expression. The TGF-β and MUC5AC mRNA expression levels were upregulated by vitamin A supplementation at a dose of 6,000 IU/kg, and the IL-13 mRNA expression level was increased at the 12,000 IU/kg supplement level. Vitamin A deficiency (control) significantly decreased the mRNA expression levels of MUC2, IgA, EGFR, IL-13 and TGF-β in trachea tissue. Histological section analysis revealed that the number of goblet cells in the tracheal epithelium was less in the 0 and 12,000 IU/kg vitamin A supplement groups than in the other groups. In conclusion, vitamin A deficiency suppressed the immunity of the airway by decreasing the IgA and mucin concentrations in neonatal chicks. This study suggested that a suitable level of vitamin A is essential for the secretion of IgA and mucin in the respiratory tract by regulating the gene expression of cytokines and epithelial growth factors.

## Introduction

In mammals and birds, immunoglobulin A (IgA) is the primary antibody secreted into mucosal cavities to serve as a first line of defense [1]. Secretory IgA (SIgA) plays an immune exclusion role against mucosal epithelium infection pathogens [2].

In the respiratory airway, SIgA plays a key role in the expansion of the immunological response to allergens or pathogenic microorganisms. In humans, the salivary SIgA concentration is related to upper respiratory tract infection [3]. The importance of SIgA in nasal anti-influenza mucosal immunity has been demonstrated by the total parenteral nutrition method to avoid influence from the gut [4]. In neonatal chicks, the immune system in the respiratory tract is vital to their health. The respiratory tract may be a largely overlooked portal of entry for *Salmonella* infections in chickens [5]. After birth, maternal antibodies gradually decrease over time. In humans, nasal secretion of SIgA is lower in healthy children than in healthy adults [6]. In domestic fowl, maternal IgA is likely to be exhausted before immune independence at 7–10 days of age [7].

Vitamin A can improve disease resistance. When dietary vitamin A is sufficient, antibody synthesis and lymphocyte proliferation in chickens are enhanced, and the morbidity and mortality caused by Newcastle disease virus and E.coli are significantly decreased [8]. In contrast, vitamin A deficiency decreases the antibody titer and bile IgA concentration after vaccination [9]. Previous studies have demonstrated the roles of vitamin A and retinoic acid (RA) receptors in T-cell differentiation and in IgA switching and production [10–14]. Vitamin A can act on B cells, which enhance humoral immunity, thereby participating in and promoting the synthesis of antibodies [15, 16]. Furthermore, vitamin A is involved in the synthesis of mucopolysaccharides among the organs' interstitium, which play an adhesion protective role in cells [17, 18]. To a certain extent, the antibody content in chickens depends on the dietary vitamin A level. In chickens fed a high dose of vitamin A in the diet, the serum antibody content is approximately 2 to 5 times higher than that in chickens not fed vitamin A [19]. Vitamin A deficiency decreases the ability to synthesize specific antibodies and weakens the lymphocyte proliferation response in broilers [20, 21]. Supplementation of vitamin A and β-carotene can strengthen the immune system for neonates [22, 23]. Moreover, the bursa of Fabricus and the thymus are impaired in chicks fed a vitamin A-free diet [24]. Vitamin A deficiency leads to epithelial squamous metaplasia and loss, which affects its integrity and density, thereby allowing the pathogen to easily invade and infect the organism [25]. Moreover, both *in vitro* and *in vivo* studies have shown that vitamin A and its derivatives are necessary for the normal growth and differentiation of epithelial cells [26–29].

Mucin glycoproteins (mucins) are the major component of airway mucus, and they provide a protective barrier against pathogenic agents. Airway mucins are mainly produced by goblet cells and submucosal gland cells [30]. Sixteen mucin genes encoding the protein backbone of mucins have been identified in the airway of humans, and mucin 5AC (MUC5AC), mucin 5B (MUC5B), and mucin 2 (MUC2) are the principal gel-forming mucins secreted in the airway [31]. The overproduction of mucins during immune challenge, however, contributes to the obstruction of the airways [32]. Vitamin A or retinoic acid has been shown to play an important role in the induction of mucin gene expression [33, 34]. In rats, the secretory mucin rMuc5AC is directly or indirectly regulated by vitamin A in the ocular surface epithelium [35]. In chickens, high concentrations of vitamin A inhibit synthesis and secretion of mucus by the chick tracheal epithelium [36]. In our preliminary experiment, IgA concentrations in serum and BALF were increased by vitamin A supplement (unpublished data, S1 Fig). Hence, we hypothesized that vitamin A supplementation is beneficial for the mature airway immune system in neonatal chicks during the first week of age.

The purpose of this study was to evaluate the effects of vitamin A supplementation on the secretion of SIgA and mucins in lungs and airways of neonatal chicks. The contents of mucin and IgA in the respiratory tract were determined, and the relevant gene expression levels were measured.

## Materials and Methods

### Experimental animals

A total of 120 healthy one-day-old broilers were obtained from a local hatchery and reared in the same environmentally controlled room. The brooding temperature was maintained at 35°C (65% relative humidity, RH) for the first 2 days and was then gradually reduced to 30°C on day 7. All of the chickens had free access to food and water during the rearing period. The experimental procedures were approved by the Institutional Animal Care and Use Committees in accordance with the criteria outlined in the Guide for the Care and Use of Laboratory Animals (Beijing, P. R. China).

### Treatment

The 120 chicks were randomly divided into 12 groups and subjected to four treatments with 3 replicates of 10 chicks from 1 day of age. The experimental chicks were given a typical corn-soybean basal diet (approximately 160 IU vitamin A/kg) supplemented with 0, 1,500, 6,000, or 12,000 IU vitamin A/kg diet in the form of vitamin A acetate (500,000 IU/g), and the basal diet was fed to the control group. The basal diet was formulated to satisfy the nutrient requirement of NRC (1994) with 3,000 Kcal metabolizable energy/kg and 21% crude protein (Table 1). Feed was provided in pellet form.

**Table 1:** Ingredients and calculated analysis of experimental diets.

Ingredients	Proportion, %
Corn	63.25
Soybean meal	29.12
Fish meal	3.0
Soybean oil	1.25
Limestone meal	1.20
Calcium phosphate	1.39
Salt	0.21
Lysine chloride, 78%	0.02
DL -Met	0.14
Choline chloride, 50%	0.20
Mineral and vitamin premix^#^	0.22
Calculated analysis	
ME, kcal/kg	3,011
CP, %	20.0
Calcium, %	0.90
Available phosphorus, %	0.45
Lysine, %	0.95
Methionine, %	0.42
Methionine + cystine, %	0.68

^#\\^Mineral and vitamin premix provided the followings per kilogram of diet: manganese, 100 mg; zinc, 75 mg; iron, 80 mg; iodine, 0.65 mg; copper, 80 mg; selenium, 0.35 mg; cholecalciferol, 0.002 MIU; vitamin E, 0.011 MIU; vitamin K, 1.0 mg; thiamine, 1.2 mg; riboflavin, 5.8 mg; niacin, 66 mg; pantothenic acid, 10 mg; pyridoxine, 2.6 mg; biotin, 0.10 mg; folic acid, 0.7 mg; and vitamin B_12_, 0.012 mg.

### Collection of BALF

At 7 days of age, three broilers at approximately the mean body weight were selected from each replicate group. Blood samples were obtained from a wing vein using a heparinized syringe. Plasma was obtained by centrifugation at 400 × g for 10 min at 4°C, and it was stored at -20°C for further analysis. After blood sampling, the chickens were sacrificed by cervical dislocation. Thereafter, the trachea and lungs were exposed, and the trachea was cannulated with a catheter and lavaged three times with 1 mL of 0.9% saline. The lavage fluid was centrifuged at 3,000 rpm for 5 min and stored at -20°C for further analysis.

### Measurement

#### IgA concentration

IgA concentration was determined with a double antibody sandwich ELISA method. The specific goat-anti-chicken IgA antibody was obtained from the American Bethyl Company (A30-103A-17). The goat anti-chicken IgA detection antibody (100 μL) was added to each well and incubated at 37°C for 1 hour. After five washes, the plates were blocked with 100 μL of PBS containing 1% BSA overnight, and the plates were then washed five times. Thereafter, 100 μL of standard or sample was added and incubated at 37°C for 1 hour. After washing, 100 μL of HRP Solution A was added and incubated at 37°C for 30 minutes. The plate was washed, and 100 μL of TMB substrate solution was added. After development at 37°C under dark conditions for 30 minutes, the reaction was stopped by adding 100 μL of stop solution. The absorbance was measured at 450 nm using a plate reader.

#### Mucin concentrations

The mucin concentrations were measured according to the phenol sulfuric acid method described by Dubois et al. [37].

#### Staining of goblet cells

Fresh trachea were fixed in 4% paraformaldehyde, dehydrated, embedded in paraffin, and sectioned using standard methods. The serial sections, which were 4-mm thick, were stained with Periodic Acid Schiff Reagent (PAS, Beijing Tian Gen Biotechnology) to evaluate the goblet cells under a light microscope. The procedure was performed as follows: i) trachea were conventionally fixed with 10% formalin and then dehydrated and embedded using standard methods; ii) sections were dewaxed with xylene; iii) sections were placed in periodate solution for 5 minutes; iv) sections were rinsed with tap water once and then rinsed twice with distilled water; v) sections were treated with Schiff’s reagent at room temperature for 15 minutes for color development; vi) sections were rinsed with tap water for 10 minutes and then rinsed with distilled water for 1 minute; vii) sections were placed in hematoxylin staining solution for 1 minute to re-dye the nucleus; viii) sections were rinsed with tap water for 15 minutes and then rinsed with distilled water to recover the blue color; ix) and sections were dehydrated in a sequential ethanol series, rinsed with xylene, and then covered with resin.

#### RNA isolation and analysis

The gene expression levels in the lung and trachea were quantified using quantitative real-time PCR. The total RNA was isolated with Trizol (Invitrogen, San Diego, CA, USA). The quality and quantity of the RNA were determined by agarose gel electrophoresis and a biophotometer (Eppendorf, Germany), respectively. RT reactions (10 μL) contained 500 ng total RNA, 5 mmol/L MgCl_2_, 1 μL of RT buffer, 1 mmol/L dNTP, 2.5 U AMV, 0.7 nmol/L oligo d (T) and 10 U ribonuclease inhibitor (TaKaRa, Dalian, China). Real-time PCR analysis was conducted using the Applied Biosystems 7500 Real-time PCR System (Applied Biosystems, Foster, CA, USA). Each RT reaction served as a template in a 20 μL PCR reaction that contained 0.2 μmol/L of each primer and SYBR green master mix (Takara, Dalian, China). The real-time PCR reactions consisted of pre-denaturation at 95°C for 10 seconds followed by 40 cycles of denaturation at 95°C for 5 seconds and annealing and extension at 60°C for 40 seconds. A standard curve was plotted to calculate the efficiency of the real-time PCR primers. β-actin was used as the housekeeping gene, and the results of the relative mRNA quantification were verified with the β-actin levels (ΔCT). The comparative CT method (2^−ΔΔCT^) was used to quantitate the mRNA expression in accordance with Wang et al [38]. The primer sequences are listed in Table 2.

**Table 2:** Gene-specific primer sequences used for gene transcription analyses of chicken.

Gene	GenBank Accession No.	Primer sequences (5’-3’)	Product size (bp)
β-actin	NM_205518.1	Forward CTGGCACCTAGCACAATGAA	123
		Reverse CTGCTTGCTGATCCACATCT	
MUC5AC	XM_003641322.2	Forward AAGACGGCATTTATTTCTCCAC	244
		Reverse TCATTACCAACAAGCCAGTGA	
MUC2	XM_001234581.3	Forward CCCTGGAAGTAGAGGTGACTG	143
		Reverse TGACAAGCCATTGAAGGACA	
IgA	S40610.1	Forward GTCACCGTCACCTGGACTACA	114
		Reverse ACCGATGGTCTCCTTCACATC	
TNF-α	HQ739087.1	Forward GAGCGTTGACTTGGCTGTC	129
		Reverse AAGCAACAACCAGCTATGCAC	
TGF-α	NM_001001614.1	Forward CCAGAAGAAGCAGACGATCA	192
		Reverse CTCTCAGTATGGCAGCAGGA	
EGFR	NM_205497	Forward CTGCCATCCAAACTGTACACGA	260
		Reverse GACCGATGCCTAGACCAACCA	
IL-6	HM179640.1	Forward CTCCTCGCCAATCTGAAGTC	152
		Reverse AGGCACTGAAACTCCTGGTC	
IL-13	NM_001007085	Forward CTGCCCTTGCTCTCCTCTGT	127
		Reverse GGGACCTGCACTCCTCTGTT	
TGF- β	M31160	Forward AGGATCTGCAGTGGAAGTGGAT	138
		Reverse CCCCGGGTTGTGTTGGT	

### Statistical Analysis

All the data were analyzed with SAS software (SAS version 8e; SAS Institute, Cary, NC, USA). A one-way ANOVA model was used to evaluate the main effect of vitamin A treatment. The data are presented as the mean ± SEM. P < 0.05 was considered statistically significant.

## Results

### Serum IgA concentration

**Fig 1:**
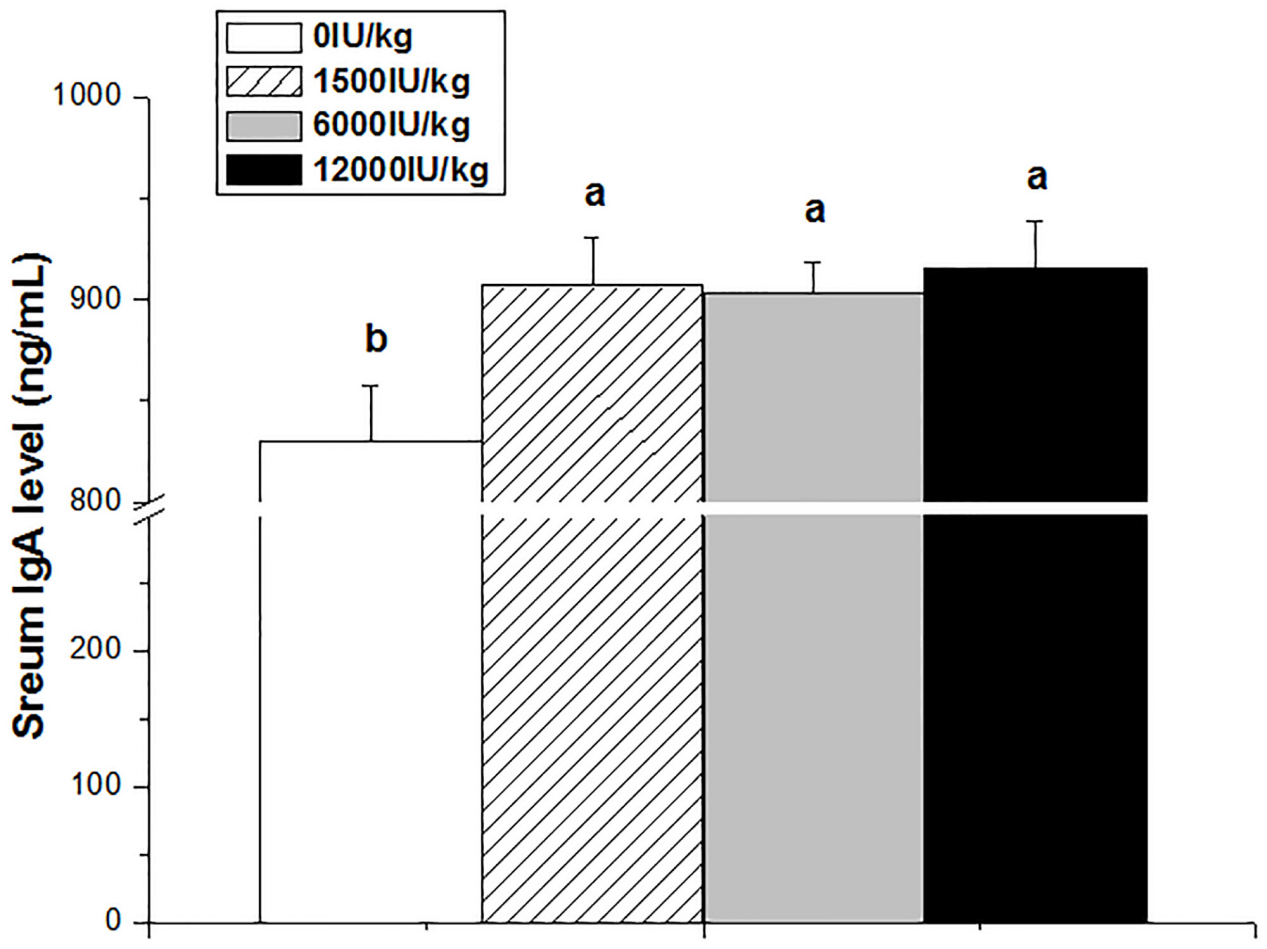
Effect of dietary vitamin A supplemental levels (0, 1500, 6000, and 12000 IU/kg) on the serum IgA concentration. Data are presented as the means ± SEM (n = 9); ^a, b, c^: Means with different letters differ significantly, *P* < 0.05.

Compared with control group, the serum IgA concentration was significantly increased by vitamin A supplementation at various levels (*P* < 0.05, Fig 1). There was no difference among different vitamin A groups (*P* > 0.05).

### Mucin and IgA concentration in the BALF

**Fig 2:**
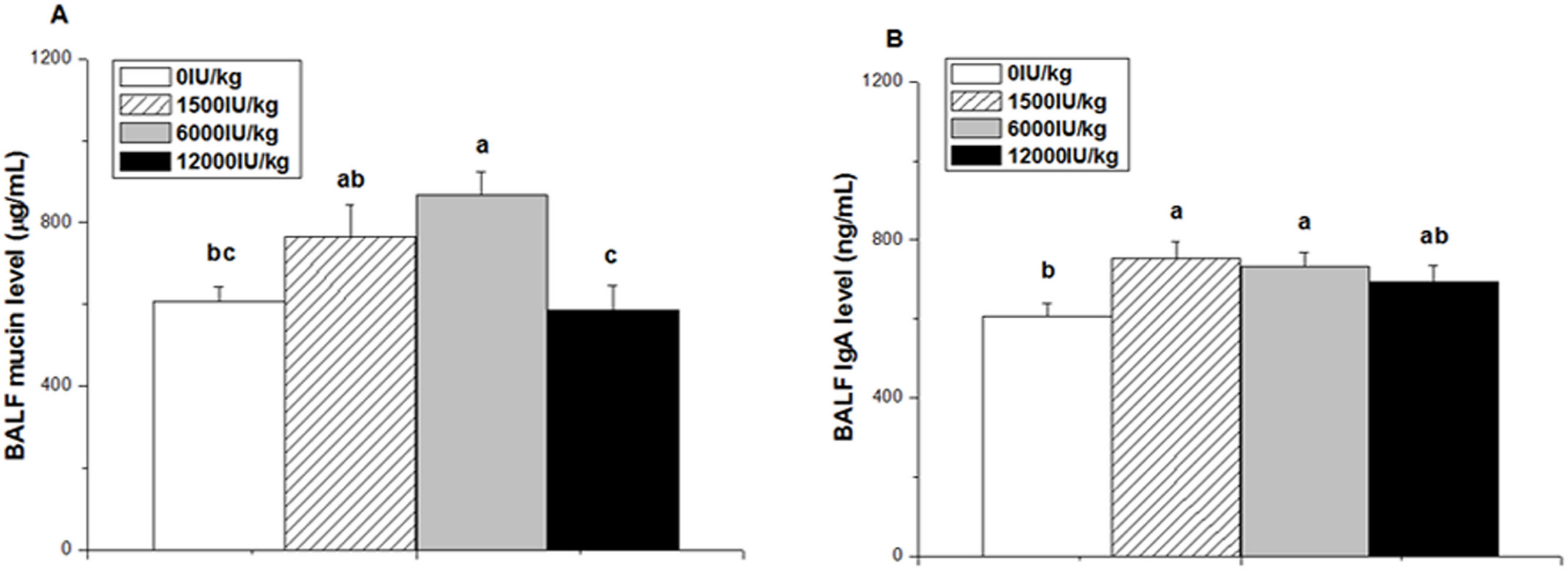
Effect of dietary vitamin A supplemental levels (0, 1500, 6000, and 12000 IU/kg) on the mucin and IgA concentrations in BALF. Data are presented as the means ± SEM (n = 9); ^a, b, c^: Means with different letters differ significantly, *P* < 0.05.

The mucin concentration in the BALF was significantly affected by dietary vitamin A supplementation (*P* < 0.01, Fig 2A). Compared with the control or 12000 IU/kg vitamin A groups, the 6000 IU/kg vitamin A group had higher mucin concentration (*P* < 0.01). However, the BALF mucin concentrations in the 1500 and 12000 IU/kg vitamin A groups were not significantly different from that of the control group (*P* > 0.05).

Compared to the control, the IgA concentration in the BALF was increased by vitamin A supplementation at the 1500 and 6000 IU/kg levels (*P* < 0.05, Fig 2B) but not at the 12000 IU/kg level (*P* > 0.05). There was no difference among different vitamin A groups (*P* > 0.05).

### Morphological observation of goblet cells

**Fig 3:**
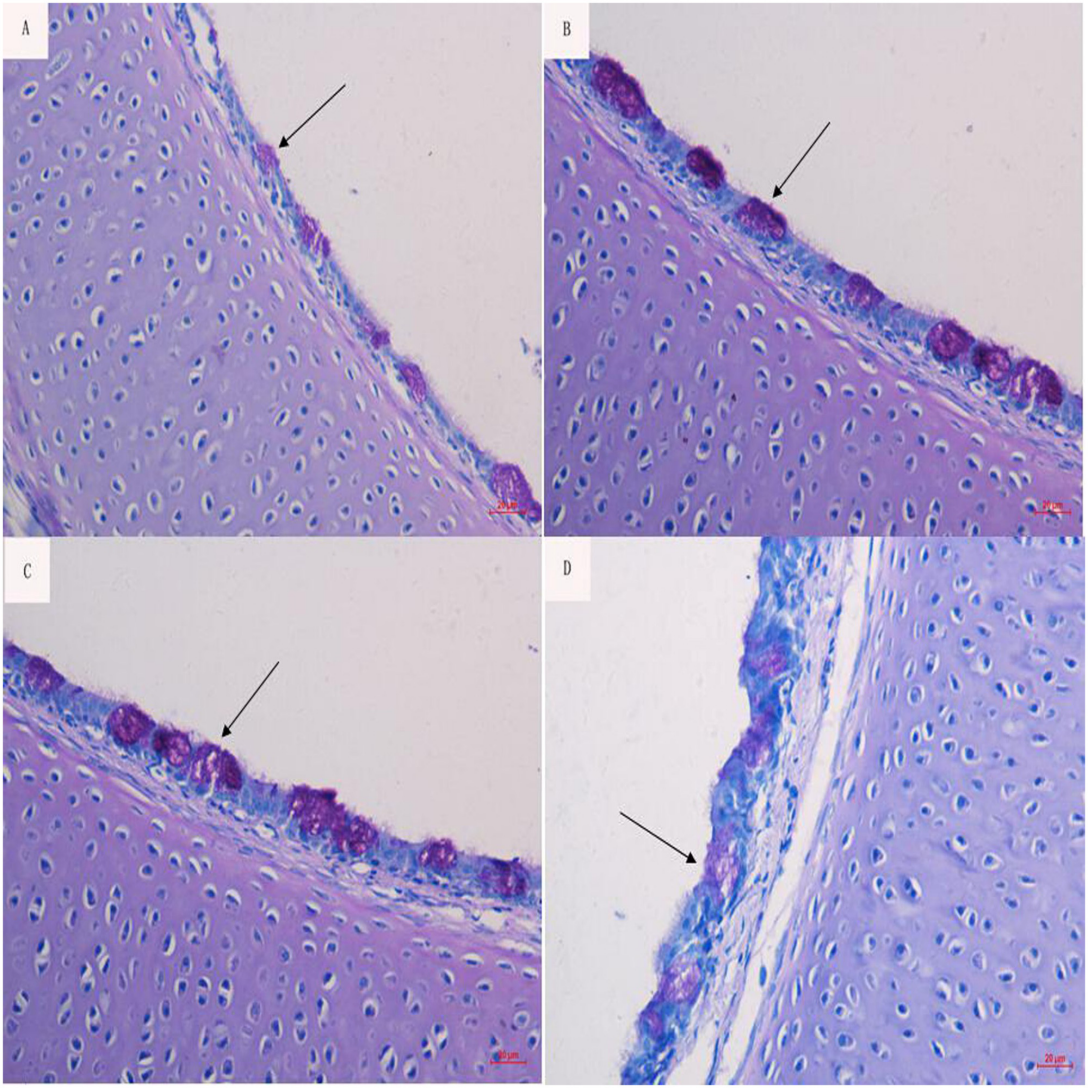
Effect of the dietary vitamin A supplemental levels (0, 1500, 6000, and 12000 IU/kg) on the production of goblet cells (black arrows). (PAS. Mic. Mag. ×400).

The number of goblet cells and the mucin contents in the control and 12,000 IU/kg vitamin A groups were less than those in the 1,500 IU/kg and 6,000 IU/kg vitamin A groups (Fig 3).

### mRNA expression levels in the lung and trachea

**Fig 4:**
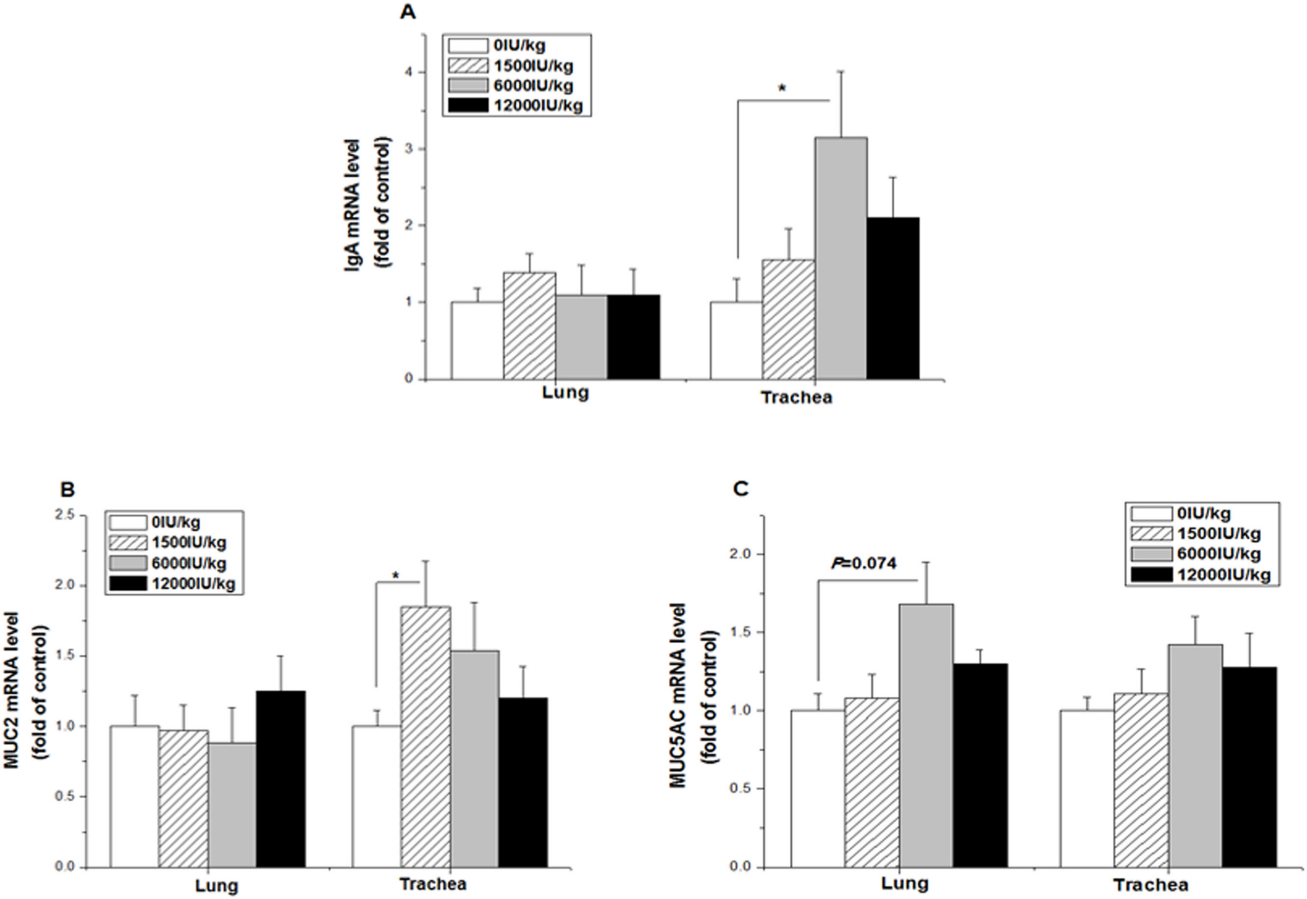
Effect of the dietary vitamin A supplemental levels (0, 1500, 6000, and 12000 IU/kg) on the IgA (A), MUC2 (B), and MUC5AC (C) mRNA expression levels in the trachea and lung tissue. Data are presented as the means ± SEM (n = 9); *: *P* < 0.05.

In the lung, the mRNA levels of IgA and MUC2 were not altered by vitamin A treatment at any supplemental level (P > 0.05, Fig 4A and 4B), whereas the mRNA level of MUC5AC tended to be increased by vitamin A supplementation at the 6,000 IU/kg level (*P* = 0.074, Fig 4C). In the trachea, however, the mRNA levels of IgA (*P* < 0.05) and MUC2 (*P* < 0.01) were upregulated by vitamin A supplementation at the 6,000 IU/kg and 1,500 IU/kg levels respectively, compared to the control group (Fig 4A and 4B). In contrast, the gene expression levels of MUC5AC were not changed by vitamin A treatment (Fig 4C).

**Fig 5:**
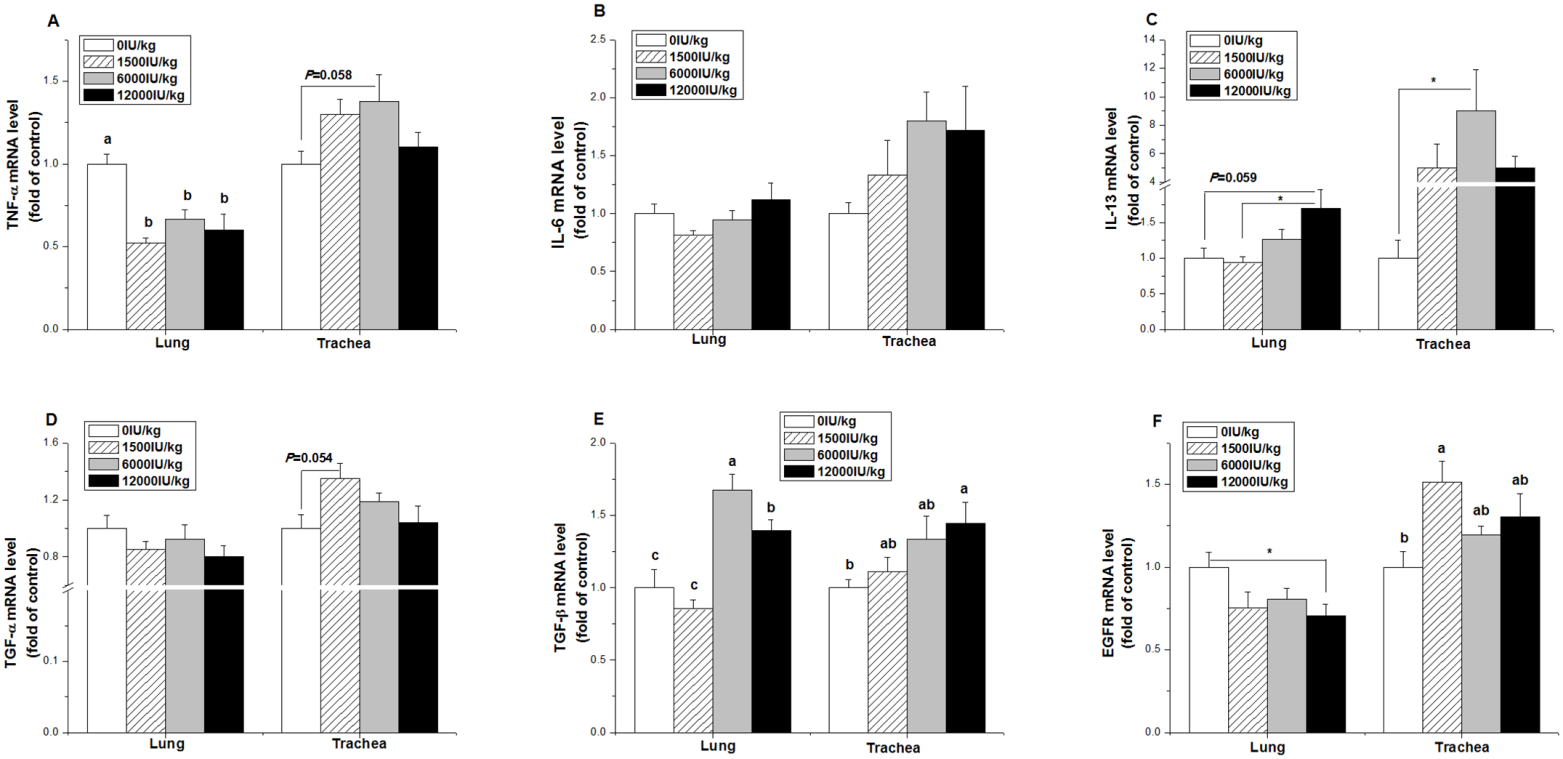
Effect of the dietary vitamin A supplemental levels (0, 1500, 6000, and 12000 IU/kg) on the TNF-α (A), IL-6 (B), IL-13 (C), TGF-α (D), TGF-β (E), and EGFR (F) mRNA expression levels in the trachea and lung tissue. Data are presented as the means ± SEM (n = 9); ^a, b, c^: Means with different letters differ significantly, *P* < 0.05; *: *P* < 0.05.

In the lung, the gene expression level of TNF-03B1 was downregulated by vitamin A supplementation at all three levels (*P* < 0.05, Fig 5A). Compared with the control, however, the mRNA level of IL-13 tended to be increased by vitamin A supplementation at the dose of 12,000 IU/kg (*P* = 0.059, Fig 5C). The expression of TGF-β was increased by vitamin A supplementation (*P* < 0.001), and the TGF-β mRNA level was higher in the 6,000 and 12000 IU/kg vitamin A treatment groups compared to the control group (*P* < 0.001, Fig 5E). In contrast, the mRNA levels of IL-6 and TGF-α were not changed by vitamin A treatment (*P* > 0.05, Fig 5B and 5D).

In the trachea, the mRNA levels of TNF-α (*P* = 0.058) and TGF-α (*P* = 0.054) tended to be respectively increased by the 6000 IU/kg and 1500 IU/kg vitamin A treatments compared to the control group (Fig 5A and 5D), whereas the gene expression of IL-6 was not influenced by vitamin A treatment (*P* > 0.05, Fig 5B). Compared with the control, the 6000 IU/kg vitamin A group had higher IL-13 mRNA levels (P < 0.05, Fig 5C). The TGF-β mRNA expression level increased with vitamin A supplementation, and the 12000 IU/kg vitamin A group had a higher TGF-β mRNA expression level than that of the control (*P* < 0.05, Fig 5E). Compared with the control group, the 1500 IU/kg group showed an upregulated EGFR mRNA level in the trachea (*P* < 0.05), but it was downregulated in the 12000 IU/kg group in the lung (*P* < 0.05, Fig 5F).

## Discussion

In this study, vitamin A supplementation improved the immunity of the respiratory tract by increasing the IgA and mucin concentrations. This study confirmed that a suitable level of vitamin A is essential for the secretion of IgA and mucin in the respiratory tract by regulating the gene expression of cytokines and epithelial growth factors.

### Vitamin A supplementation improves IgA concentrations of neonatal chicks

Previous studies have suggested a possible regulatory role rather than a constitutive role for vitamin A in immune responsiveness [20, 21]. Vitamin A stimulates the development and differentiation of B lymphocytes [39]. Vitamin A can stimulate the intestinal tract to produce SIgA and Th2 cytokines in both malnourished and normal mice, enhancing the immune function of the intestinal mucosa [40].

In humans and mice, SIgA plays a key role in the immunological response of the respiratory airway to allergens or pathogenic microorganisms [3, 4]. In an influenza virus infection BALB/C mouse model, vitamin A can promote the generation of specific antibodies and the production of specific SIgA and Th2 cytokines when there is an acute lower respiratory tract infection [41]. The number of IgA-secreting plasma cells in the salivary glands is significantly less in a vitamin A-deficient group than in a control group [42]. In accordance with the reports in mammals, the present study showed that IgA concentrations in serum and BALF were improved by vitamin A supplementation in neonatal chicks, suggesting that vitamin A supplementation enhances the local specific mucosal immune system in the respiratory tract. In poultry, the respiratory tract is an important infection route for pathogenic organisms [5]. In neonatal chicks, the development of the immune system is vital to their health, especially as the maternal IgA is likely to be exhausted before immune independence at 7–10 days of age [7]. In this experiment, there was no difference in feed consumption (approximately 20.5 g/d) among the different vitamin A treatments, indicating that feed consumption is not responsible for the difference caused by vitamin A treatment. Hence, this result implied that vitamin A supplementation at an adequate level is relevant to the immunity of neonatal chicks. The upregulated gene transcription level for IgA in the trachea but not in the lung may imply that the regulatory effect of vitamin A on SIgA secretion is tissue specific.

The beneficial effect of vitamin A at a moderate level (6000 IU/kg) is reversed by a high dose of vitamin A supplementation (12000 IU/kg). In humans, high dose vitamin A supplements cause modest adverse effects in children recovering from pneumonia [43]. However, this result does not agree with the work of Cui et al. [41], who reported that high dose vitamin A supplements may enhance Th2-mediated immune responses in mice. In this study, the mRNA levels of IL-6 and IL-13 were differentially influenced by high dose vitamin A supplementation. Hence, the effect of high dose vitamin A supplementation needs to be investigated further.

In neonatal chicks, the persistence of maternal IgA in the gut is enabled by goblet cell uptake and consequent release in a mucin-like layer on enterocyte apical surfaces [7]. In this study, the abundance of goblet cells in airways was improved in the 1,500 and 6,000 IU/kg vitamin A supplement groups compared to the 0 and 12,000 IU/kg groups, suggesting that vitamin A deficiency or vitamin A excess is a disadvantage for the proliferation and differentiation of mucosa cells. This result was supported by the observation of Friedman et al., who reported that the reduction in resistance to E. coli infection resulting from vitamin A excess or deficiency may have been compounded by a delayed immune response [8]. However, the beneficial effect of goblet cells on IgA persistence in airways needs to be further investigated.

### Vitamin A supplementation improves airway mucin secretion in neonatal chicks

Mucins are the major component of airway mucus and are present in all wet-surface mucosal epithelia [44]. Vitamin A and its metabolite, retinoic acid, are necessary for maintaining mucosal cell differentiation, mucin production, and mucin gene expression [45–47]. Vitamin A deficiency impairs mucin production. The gene expression level of mucin in a rat tracheal organ culture was barely detectable in the absence of retinoic acid [17]. In line with previous studies, the increased BALF mucin concentration by vitamin A supplementation at a dose of 6,000 IU/kg compared to the control indicated that vitamin A supplementation at a moderate level improves mucin secretion. Compared to the moderate level, however, high dose vitamin A supplementation suppressed mucin secretion, which agreed with the result of Aydelotte, who reported that high concentrations of vitamin A inhibit synthesis and secretion of mucus by the chick tracheal epithelium [36]. These results suggest that vitamin A deficiency or high dose vitamin A is disadvantageous for mucin secretion.

Airway mucins are produced mainly by goblet cells and submucosal gland cells [30]. Our study observed that the goblet cell number in airways was increased by vitamin A supplementation at moderate levels (1500 to 6000 IU/kg). A similar phenomenon has also been observed in the intestinal epithelium, in which vitamin A deficiency causes mucosa damage and a decrease in the number of mucosa goblet cells [25]. Vitamin A deficiency causes hyperproliferation of enterocytes and decreases the maturation of cells in the small intestinal mucosa [48]. The modified maturation and differentiation of the small intestinal mucosa by vitamin A deficiency occurs at both the transcriptional and post-transcriptional levels [49].

In humans, 16 mucin genes in total have been identified in the airway. Among these genes, MUC5AC, MUC5B, and MUC2 are the principal gel-forming mucins secreted in the airway [31]. In chickens, there are obvious homologues of the primate and rodent MUC2, MUC5AC, MUC5B, and MUC6 genes both with respect to sequence of the VWD domains as well as to their localization and direction in the gene cluster [50]. In this study, the MUC2 and MUC5AC expression levels in the lung and trachea were influenced by vitamin A supplementation, suggesting that vitamin A is involved in the regulation of mucin gene transcription in neonatal chicks. This result was in accordance with mammal studies. Vitamin A or retinoic acid has been shown to play an important role in the induction of mucin gene expression [33, 34]. In vitro cultured airway epithelial cells have higher MUC5AC expression in the presence of vitamin A [51, 52]. In human tracheobronchial epithelial cells, the addition of RA restores mucous differentiation and induces the expression of the mucin genes MUC2, MUC5AC, and MUC5B [33]. RA activates cyclic AMP response element-binding protein (CREB) to stimulate MUC5AC expression and mucous differentiation in primary bronchial epithelial cells [34]. In this study, the altered MUC2 and MUC5AC gene expression was in line with the result of the BALF mucin concentration.

### Vitamin A influences the expression of cytokines and growth factors

Naive, uncommitted T helper precursor cells (Th) can differentiate into Th1 or Th2 cells [53]. Th1 cells secrete IFN-γ, TNF-α, and TNF-β, and they promote delayed type hypersensitivity reactions. Moreover, Th2 cells produce mainly IL-4 and IL-5, and they promote humoral and allergic responses [53]. Vitamin A deficiency can lead to a change in the proportion of the T cell subsets [54]. Recently, it has been shown that RA acts as a cofactor of TGF-β in regulatory T cell generation [55]. Furthermore, the vitamin A metabolite, retinoic acid, has been shown to be a key regulator of TGF-β-dependent immune responses, and it has been shown to inhibit IL-6 and promote anti-inflammatory regulatory T cell differentiation [56]. In this study, vitamin A supplementation increased TGF-β expression both in lung and trachea tissues, suggesting that vitamin A plays a role in TGF-β gene expression in the airway of neonatal chicks. Meanwhile, TGF-β is associated with IgA switching and secretion [57]. The IgA-inducing effect of RA partially depends on TGF-β [58, 59]. Collectively, these results imply that vitamin A supplementation facilitates T cell differentiation and promotes IgA secretion.

Vitamin A supplementation had no promoting effect on TGF-α expression, which was the opposite result compared to mammals. The expression of TGF-α is closely associated with the development of squamous epithelium, and vitamin A can effectively reverse this phenotype by downregulating the expression of TGF-α at the mRNA level [60]. The role of TGF-α in chicks needs to be further investigated.

The proliferation of respiratory epithelium needs a number of growth factors such as the epidermal growth factor (EGF). EGF exerts the regulatory effect by binding to its receptor EGFR, which is located in the cell membrane [61]. The stimulation of EGFR by its ligands, namely EGF and TGF-α, enhances MUC5AC mucin expression in airway epithelial cells [62, 63]. EGFR activation results in goblet cell proliferation and increased MUC5AC gene and protein expression [64]. The effect of EGF on mucous cell differentiation and gene expression is dependent on species and vitamin A dose. In cultured rat tracheal epithelial cells, EGF enhances mucous cell differentiation and mucin gene expression [65]. In contrast, EGF inhibits mucin secretion and MUC5AC expression in human bronchial epithelial cell cultures in the absence or low concentration of RA [66]. However, the suppressive effect of EGF on mucin secretion is rescued by higher concentrations of RA [66]. In this study, high dose vitamin A supplementation suppressed the EGFR transcriptional level in the lung rather than in the trachea. Collectively, these results imply that the EGFR signaling pathway is involved in the regulatory effect of vitamin A on cell proliferation.

As a proinflammatory cytokine, TNF-α plays an important role in the differentiation of epithelial cells. TNF-α stimulates MUC5AC expression in human airway epithelial cells [67]. Moreover, TNF-α induces mucin hypersecretion and MUC2 gene expression in human airway epithelial cells [68]. An RA receptor-α antagonist inhibits the TNF-α stimulation of MUC2 and MUC5AC mRNA expression [69]. In this study, the vitamin A-induced downregulation of TNF-α mRNA levels in the lung suggested that vitamin A suppresses the TNF-α-induced immune response in neonatal chick lungs. These findings agreed with the results in mice reported by Cui et al., who demonstrated that vitamin A decreases the production of IFN-g, an important Th1 cytokine [41]. Moreover, the different changes in TNF-α mRNA levels in lung and trachea tissues indicated that vitamin A regulates the gene expression of TNF-α in a tissue-dependent manner.

As a pleiotropic cytokine produced in large quantities by activated CD4+Th2 lymphocytes, IL-13 functions as a potent inducer of airway epithelial cell hypertrophy and goblet cell hyperplasia [70]. Vitamin A and the Th2 cytokines play important roles in the induction of mucin gene expression and mucus hypersecretion. IL-13 exposure increases MUC5AC and MUC2 mRNA expression in goblet transition cells [71]. In the present study, the upregulated IL-13 expression resulting from vitamin A treatment indicated that vitamin A promotes airway immune function via the IL-13 pathway. Moreover, the IL-4/13 Th2 cytokines and RA both can alter the activity of enzymes that synthesize the branching mucin carbohydrate structure in airway epithelial cells, potentially leading to altered mucin carbohydrate structure and properties [72, 73].

## Conclusions

In conclusion, vitamin A deficiency suppressed the airway immunity by decreasing BALF IgA and mucin concentrations in neonatal chicks. These results suggested that vitamin A supplementation at an appropriate level can improve the immunity of the respiratory tract by stimulating gene expression of cytokines and epithelial growth factors.

### Ethics statement

The present study was approved by Shandong Agricultural University and conducted according to the Guidelines for Experimental Animal Research of the Ministry of Science and Technology (Beijing, China).

## Supporting Information

S1 ARRIVE Checklist ARRIVE Checklist. (DOC)

S1 Fig Effect of vitamin A supplementation (0, 1500, 3000, and 6000 IU) on IgA and TNF-α concentrations in serum (A) and BALF (B), and immune organ index (C) in chicks. (DOC)
