# Supplementary material for: Vitamin A Deficiency Impairs Mucin Expression and Suppresses the Mucosal Immune Function of the Respiratory Tract in Chicks
Source: PLoS One. 2015 Sep 30;10(9):e0139131. doi: 10.1371/journal.pone.0139131 (PMC4589363; doi:10.1371/journal.pone.0139131)
Supplement: S1 ARRIVE Checklist — (DOC) [file pone.0139131.s001.doc]

The ARRIVE Guidelines Checklist

Animal Research: Reporting In Vivo Experiments

|  | ITEM | RECOMMENDATION | Section/  Page number |
| --- | --- | --- | --- |
| Title | 1 | Vitamin A Deficiency Impairs Mucin Expression and Suppresses the Mucosal Immune Function of the Respiratory Tract in Chicks | 1 |
| Abstract | 2 | The immune system is immature at time of hatch in chicken. The development of respiratory immune system after hatching is vital to the young chicks. The aim of this study was to investigate the effect of dietary vitamin A supplement levels on respiratory mucin and IgA production in chicks. 120 day-old broiler chicks were randomly divided into 4 groups consisting of 3 replicates of 10 broilers and subjected to dietary vitamin A supplement levels of 0, 1,500, 6,000 and 12,000 IU/kg for 7 days. Compared with control birds, vitamin A supplementation significantly increased the mucin and IgA levels in the BALF and IgA level in serum. In the lungs, vitamin A supplementation down-regulated the TNF-α and EGFR mRNA expression. Vitamin A supplementation at a dose of 6,000 IU/kg up-regulated TGF-β and MUC5AC mRNA expression while the IL-13 mRNA expression was increased by the 12,000 IU/kg supplement level. Vitamin A deficiency (no supplement) can significantly decrease the mRNA expression of MUC2, IgA, EGFR, IL-13 and TGF-β in trachea tissue. Histological section analysis revealed that the number of goblet cells in the tracheal epithelium was significantly lower in the 0 and 12,000 IU/kg vitamin A supplement groups. In conclusion, vitamin A deficiency suppressed the immunity of airway by decreasing the IgA and mucin concentrations in neonatal chicks. The result suggests that vitamin A supplementation improves the immunity of the respiratory tract through the direct effect of vitamin A and by stimulating gene expression of cytokines and epithelial growth factors. | 2 |
| INTRODUCTION |  |  |  |
| Background | 3 | a. In mammals and birds, immunoglobulins A (IgA) is the primary antibody secreted into mucosal cavities to serve as a first line of defense. IgA-dependent mucosal immunity and nonspecific innate immune factors (such as the mucociliary clearance system) collaborate with each other against foreign objects. Secretory SIgA (SIgA) plays an immune exclusion role against mucosal epithelium infection pathogens. The SIgA's major role as a first line of immune defense has been recognized in gut.  In respiratory airway, SIgA play a key role in the elaboration of the immunological response to allergens or pathogenic microorganisms. In human, salivary SIgA concentration is related to upper respiratory tract infection. The importance of SIgA in nasal anti-influenza mucosal immunity was demonstrated with the total parenteral nutrition method to avoid the influence from gut. In neonatal chicks, the immune system in respiratory tract is vital to their health. The respiratory tract may be a largely overlooked portal of entry for *Salmonella* infections in chickens. After birth, maternal antibody gradually decreased over time. In human, nasal secretion SIgA mean value is lower in the healthy child than in the healthy adult. In domestic fowl, maternal IgA is likely to be exhausted before immune independence at 7-10 days of age.  Vitamin A can improve disease resistance. When dietary vitamin A is sufficient, the ability to synthesize antibodies and the lymphocyte proliferation reactions in chickens are enhanced, and meanwhile the morbidity and mortality caused by Newcastle disease virus and E. coli are significantly decreased. Previous studies have demonstrated the roles of vitamin A and RA receptors in T-cell differentiation and of IgA switching and production. Vitamin A can act on B cells, enhancing humoral immunity, participating in and promoting the synthesis of antibodies. Furthermore, vitamin A is involved in the synthesis of mucopolysaccharides among the organs’ interstitium, which play an adhesion protective role in cells. To a certain extent, the antibody content in chickens depends on the vitamin A dose in the diet. In chickens fed a high dose of vitamin A in the diet, the antibody content in the serum is 2 to 5 times higher than that in chickens not given vitamin A. A dietary vitamin A deficiency decreases the ability to synthesis specific antibodies and weakens the lymphocyte proliferation response in broilers. Supplementation of vitamin A and β-carotene can strengthen the immune system for neonates. Moreover, the bursa of Fabricus and the thymus were impaired in chicks fed a vitamin A-free diet and A partial vitamin A deficiency adversely affected the relative bursa weight. Vitamin A deficiency leads to epithelial squamous metaplasia and loss, which affects its integrity and density, thereby allowing the pathogen to easily invade and infect the organism. Moreover, both in vitro and in vivo studies have shown that vitamin A and its derivatives are necessary for the normal growth and differentiation of epithelial cells.  Mucin glycoproteins (mucins) are the major component of airway mucus and provide a protect barrier against pathogenic agents. Airway mucins are produced mainly by goblet cells and submucosal gland cells. There are 16 mucin genes that encode protein backbone of mucins have been identified in the airway of human and mucin 5AC (MUC5AC), mucin 5B (MUC5B), and mucin 2 (MUC2) are the principal gel-forming mucins secreted in the airway. The overproduction of mucins during immune challenge, however, contributes to the airway obstruction of the airways. Vitamin A or retinoic acid has been proved to play an important role in the induction of mucin gene expression.  b. At time of hatch, the immune system is immature in neonatal chick. Hence, we hypothesized that vitamin A supplementation is beneficial for the mature of airway immune system in neonatal chicks during the first week of age. | 3-5 |
| Objectives | 4 | The purpose of this study was to evaluate the effects of vitamin A supplementation on the secretion of SIgA and mucins in lung and airways of neonatal chicks. The contents of mucin and IgA in respiratory tract were determined and the relevant gene expressions were measured as well. | 5 |
| METHODS |  |  |  |
| Ethical statement | 5 | The present study was approved by Shandong Agricultural University and conducted according to the Guidelines for Experimental Animal Research of the Ministry of Science andTechnology (Beijing, China). | 28 |
| Study design | 6 | a. The number of sample is 9 in every group.  b. The 120 chicks were randomly divided into 12 groups and subjected to four treatments; 3 replicates of 10 chicks for each treatment.  c. Use a single chick as experimental units. | 6 |
| Experimental  procedures | 7 | a. The experimental chicks were given a corn-soybean basal diet supplemented with 0, 1,500, 6,000 and 12,000 IU vitamin A/kg diet. The chickens were sacrificed by cervical dislocation.  b. At 7 days of age.  c. rearing cage. | 5-6 |
| Experimental  animals | 8 | a. Polyculture broiler chickens (Arbor Acres, Gallus gallus domesticus). Each chick weighed 165 grams  approximately at 7 days.  b. Broilers were obtained from a local hatchery. The animals is healthy without gene modification, being immuning and carrying out experiment previously. | 5 |
| Housing and husbandry | 9 | 1. Brood cage. 10 chicks in a same cage as each treatment.   b. The experimental chicks were given a corn-soybean basal diet. The basal diet was designed to include 3,000 Kcal metabolizable energy/kg and 21% crude protein. All of the other nutrients were formulated exceeding the recommendations by the NRC. All of the feeds were provided as pellets. The brooding temperature was maintained at 35ºC (65% RH) for the first 2 days and was then gradually reduced to 30ºC on day 7. All of the chickens had free access to food and water during the rearing period. The experimental procedures were approved by the Institutional Animal Care and Use Committees in accordance with the criteria outlined in the Guide for the Care and Use of Laboratory Animals (Beijing, P. R. China). | 5-6 |
| Sample size | 10 | a. The 120 chicks were randomly divided into 12 groups and subjected to four treatments; 3 replicates of 10 chicks for each treatment.  b. At 7 days of age, three broilers at approximately the mean body weight were selected from each replicate group. Hence, the number of sample is 9. | 6 |
| Allocating  animals to  experimental  groups | 11 | a. The chicks were randomly divided into four treatments: 0, 1,500, 6,000 and 12,000 IU vitamin A/kg diet.  b. Experiment animals in every group were treated at the same time. | 6 |
| Experimental outcomes | 12 | **Serum IgA concentration**  When the vitamin A supplement was 0 IU/kg, the IgA concentration in the serum was significantly lower than in the other groups. There were no differences among the other groups.  **Mucin and IgA concentration in the BALF**  Mucin concentration in respiratory tract were significantly affected by dietary vitamin A supplementation. The 6000 IU/kg vitamin level group had the highest mucin concentrations compared with the other groups, whereas the control the 12000 IU/kg group had the lowest ones.  The concentration of IgA in the control group was lower compared with the 1,500 IU/kg and 6,000 IU/kg vitamin A groups, but there was no significant difference compared with the 12,000 IU/kg groups.  **Morphological observation on Goblet cell**  The number of goblet cells and the mucin contents in control and 12,000 IU/kg dietary vitamin A supplemented group were less than those in 1,500 IU/kg and 6,000 IU/kg vitamin A groups.  **mRNA expression levels in lung and trachea**  The mRNA levels of IgA and MUC2 were increased by vitamin A supplementation in trachea rather than in lung. In contrast, the gene expression levels of MUC5AC tended to be upregulated by vitamin A supplementation at 6,000 IU/kg in lung.  In lung, the gene expressions of TNF-α were down-regulated by vitamin A supplementation at all the three levels. In contrast, the expression of IL-13 and TGF-β were increased by vitamin A supplementation. The gene expression of IL-13 tended to be increased by vitamin A supplementation at a dose of 12,000 IU/kg. The chicks in 12000 IU/kg vitamin A group had higher mRNA level of IL-13 than that in control and 1500 IU/kg group. The TGF-β mRNA expression was increased by 6,000 and 12000 IU/kg vitamin A treatments. However, the mRNA levels of IL-6 and TGF-α were not changed by vitamin A treatment.  In trachea, the mRNA levels of TNF-α and TGF-α tended to be respectively increased by 6000 IU/kg and 1500 IU/kg vitamin A treatment, whereas the gene expression of IL-6 was not influenced by vitamin A treatment. Compared with control, 6000 IU/kg vitamin A group had higher IL-13 mRNA levels. The TGF-β mRNA expression level increased with vitamin A supplementation and the 12000 IU/kg vitamin A group had higher level than that of control. Compared with control group, the EGFR mRNA level was upregulated in 1500 IU/kg group in trachea, but down-regulated in 12000 IU/kg chicks in lung. | 9-11 |
| Statistical methods | 13 | a. All the data were analyzed with SAS software (SAS version 8e; SAS Institute, Cary, NC, USA). A one-way ANOVA model was used to evaluate the means among various groups. The data are shown as the mean ± SEM. P < 0.05 was considered statistically significant.  b. The analysis unit of each data set is single animal. | 9 |
| RESULTS |  |  |  |
| Baseline data | 14 | The neonatal chicks is healthy and weigh 40g approximately . |  |
| Numbers  analysed | 15 | a. The number of sample is 9 in every group. | 26-27 |
| Outcomes and  estimation | 16 |  |  |
| Adverse events | 17 | No adverse events. |  |
| DISCUSSION |  |  |  |
| Interpretation/  scientific implications | 18 | Vitamin A improved the immunity of the respiratory tract by increasing the IgA and mucin concentrations.  **Vitamin A supplementation improved IgA concentrations of neonatal chicks**  Previous studies have suggested a possible regulatory role rather than a constitutive role for vitamin A in immune responsiveness. Vitamin A stimulates the development and differentiation of B lymphocytes. Vitamin A can stimulate the intestinal tract to produce SIgA and Th2 cytokines in both malnourished and normal mice, enhancing the immune function of the intestinal mucosa.  Whether in mice or in human, SIgA play a key role in the immunological response of respiratory airway to allergens or pathogenic microorganisms. In an influenza virus infection BALB/C mouse model, vitamin A can promote the generation of specific antibodies and the production of specific SIgA and Th2 cytokines when there is an acute lower respiratory tract infection. The number of IgA secreting plasma cells in the salivary glands was significantly less in the vitamin A deficient group than in the control group. In consistent with the study in mammals, the present study showed that IgA concentrations in serum and BALF were improved by vitamin A supplementation in neonatal chicks, suggesting that vitamin A supplementation enhances the local specific mucosal immune in respiratory tract. In poultry, respiratory tract is an important infection route of pathogenic organism. In neonatal chicks, the development of immune system is vital to their health, especially as the maternal IgA is likely to be exhausted before immune independence at 7-10 days of age. Hence, the result implies that vitamin A supplementation at adequate level is relevant to the health of neonatal chicks.  In neonatal chicks, the persistence of maternal IgA in the gut was enabled by goblet cell uptake and consequent release in a mucin-like layer on enterocyte apical surfaces. In this study, the abundance of goblet cells in airways was improved by vitamin A supplementation. However, the beneficial effect of goblet cells on IgA persistence in airways needs to be investigated further.  **Vitamin A supplementation improved airway mucin secretion in neonatal chicks**  Mucins are the major component of airway mucus and present in all wet-surface mucosal epithelia. Vitamin A and its metabolite retinoic acid are necessary for maintaining mucosal cell differentiation, mucin production and mucin gene expression. Vitamin A deficiency impairs the production of mucins. The gene expression level of mucins in a rat tracheal organ culture was barely detectable in the absence of retinoic acid. In line with the previous studies, the increased BALF mucin concentration by vitamin A supplement at a dose of 6,000 IU/kg compared to control indicated that vitamin A supplementation improved mucin secretion.  Airway mucins are produced mainly by goblet cells and submucosal gland cells. Our study observed that the goblet cell number in airways was increased by vitamin A supplementation at moderate levels (1500 to 6000 IU/kg). The same effect has also been observed in the intestinal epithelium, in which a vitamin A deficiency could cause digestive tract mucosa damage and a decrease in the number of mucosa goblet cells. Vitamin A deficiency inhibits the production and secretion of total mucus, perhaps by significantly reducing the MUC5AC mRNA expression in the lungs and the MUC2 expression in the trachea tissue as well as reducing the number and altering the morphology of goblet cells.  In human, 16 mucin genes in total have been identified in the airway. Among of them, MUC5AC, MUC5B, and MUC2 are the principal gel-forming mucins secreted in the airway. In the chicken, there were obvious homologues of the primate and rodent Muc2, Muc5ac, Muc5b, and Muc6 both with respect to sequence of the VWD domains as well as to their localization and direction in the gene cluster. In this study, MUC2 and MUC5AC expression in lung and trachea were respectively influenced by vitamin A supplementation, suggesting vitamin A is involved in the regulation of mucin gene transcription in neonatal chicks. This result was in accordance with the works in mammals. Vitamin A or retinoic acid has been proved to play an important role in the induction of mucin gene expression. In vitro cultured airway epithelial cells, the expression of MUC5AC expression is higher in the presence of vitamin A. In human tracheobronchial epithelial cells, the addition of RA restored mucous differentiation and induced the expression of the mucin genes MUC2, MUC5AC, and MUC5B. Except of the canonical RA receptor (RAR)/retinoid X receptors (RXRs) pathway, RA could rapidly activated cyclic AMP response element-binding protein (CREB) via a nonclassical pathway, the protein kinase C (PKCɑ)/Raf/ERK kinase (MEK)/ extracellular signal-regulated kinase (ERK)/p90 ribosomal S6 kinase (RSK), to stimulate MUC5AC expression and mucous differentiation in primary bronchial epithelial cells. The two pathways cooperatively facilitate the differentiation of bronchial epithelia. In this study, MUC2 and MUC5AC expression levels were differently affected in lung and trachea by vitamin A treatment, the underlying mechanism needs to be studied further. This study used broilers as an *in vivo* model to confirm that vitamin A is an essential nutrient for regulating mucin gene expression and secretion in respiratory tract.  **Vitamin A influences the expression of cytokines and growth factors**  The naive, uncommitted T helper precursor cells (Th) can differentiate into Th1 or Th2 cells. Th1 cells secrete IFN-γ, TNF-α, and TNF-β and promote delayed type hypersensitivity reactions, whereas Th2 cells produce mainly IL-4 and IL-5, and promote humoral and allergic responses. Vitamin A deficiency can lead to a change in the proportion of the T cell subsets. Recently, it was proved that RA could act as a cofactor of TGF-β in regulatory T cell generation. Furthermore, vitamin A metabolite retinoic acid has been proved to be a key regulator of TGF-β-dependent immune responses, capable of inhibiting the IL-6 driven induction of proinflammatory IL-17 cytokine-producing T helper 17 (Th17) cells and promoting anti-inflammatory regulatory T cell differentiation. In this study, vitamin A supplementation increased TGF-β expression both in lung and trachea tissues, suggesting that vitamin A play a role in TGF-β gene expression in the airway of neonatal chicks. TGF-β is associated with IgA switching and secretion. The IgA-inducing effect of RA partially depends on TGF-β. Collectively, the result implies that vitamin A supplementation facilitates T cell differentiation and promotes IgA secretion.  This study showed that vitamin A supplementation had no promoting effect on TGF-ɑ expression, except of a trend of increase at the dose of 1500 IU/kg. This result was not in line with the result in mammals. The expression of TGF-ɑ is closely associated with the development of squamous epithelium and that vitamin A can effectively reverse this phenotype by downregulating the expression of this gene at the mRNA level.  The proliferation of respiratory epithelium needs a number of growth factors such as the epidermal growth factor (EGF). EGF takes the regulatory effect by binding to its receptor EGFR, located in the cell membrane. The stimulation of EGFR by its ligands, EGF and TGF-α, causes MUC5AC mucin expression in airway epithelial cells. EGFR activation resulted in goblet cell proliferation and increased MUC5AC gene and protein expression. In this study, the down-regulated level in lung but upregulated level in trachea for EGFR gene expression by vitamin A supplementation indicated that vitamin A is involved in the EGFR signaling pathway. The effect of EGF on mucous cell differentiation seems to be species dependent. In cultured rat tracheal epithelial cells, EGF enhanced mucous cell differentiation and mucin gene expression. In contrast, EGF inhibited mucin secretion and MUC5AC expression in human bronchial epithelial cell cultures. However, the suppressive effect of EGF on mucous phenotype was rescued by higher concentrations of RA. Collectively, the present result may imply that the effect of vitamin A is tissue specific.  As a proinflammatory cytokine, TNF-α plays an important role in the differentiation of epithelial cells. TNF-ɑ stimulates MUC5AC expression in human airway epithelial cells. Moreover, TNF-α induces mucin hypersecretion and MUC2 gene expression by human airway epithelial cells. In this study, the downregulated TNF-ɑ mRNA level in lung by vitamin A treatment suggests that vitamin A could regulate TNF-ɑ signal in chick. RA receptor-α antagonist could inhibit the stimulation of TNF-α on MUC2 and MUC5AC mRNA expression. The result may imply that vitamin A suppressed the TNF-ɑ induced immune response in lung of neonatal chicks. Moreover, the conflict change in TNF-ɑ mRNA levels in lung and trachea tissues indicated that vitamin A could regulate the gene expression of TNF-ɑ in a tissue dependent manner.  As a pleiotropic cytokine produced in large quantities by activated CD4+Th2 lymphocytes, IL-13 could function as a potent inducer of airway epithelial cell hypertrophy and goblet cell hyperplasia [64]. Vitamin A and the T helper 2 cytokines (IL-4 and IL-13) both play important roles in the induction of mucin gene expression and mucus hypersecretion. IL-13 exposure increased MUC5AC and MUC2 mRNA expression in goblet transition cells. In the present study, the upregulated IL-13 expression by vitamin A treatment indicated that vitamin A promote airway immune function via IL-13 pathway. Moreover, it was proved that IL-4/13 T helper 2 cytokines and RA both can alter the activity of enzymes that synthesize branching mucin carbohydrate structure in airway epithelial cells, potentially leading to altered mucin carbohydrate structure and properties. | 11-17 |
| Generalisability/  translation | 19 | This study provides a theoretical basis for the important role of vitamin A in the newborn respiratory tract development and immune system enhancement at period of pregnancy or infant early development. |  |
| Funding | 20 | This research was supported by the National Natural Science Foundation of China (31272467, 31472114). | 28 |
