# Supplementary material for: Vitamin A Deficiency Impairs Mucin Expression and Suppresses the Mucosal Immune Function of the Respiratory Tract in Chicks
Source: PLoS One. 2015 Sep 30;10(9):e0139131. doi: 10.1371/journal.pone.0139131 (PMC4589363; doi:10.1371/journal.pone.0139131)

**Sl Supporting information**

**S1** Effect of vitamin A supplementation on Ig A and TNF-ɑ concentrations, and immune organ index in chicks

S1-Fig 1 Effect of vitamin A supplementation (0, 1500, 3000, and 6000 IU) on Ig A and TNF-ɑ concentrations, and immune organ index in chicks

Data are presented as the means ± SEM (n=9); a, b, c: Means with different letters differ significantly, *P* < 0.05.


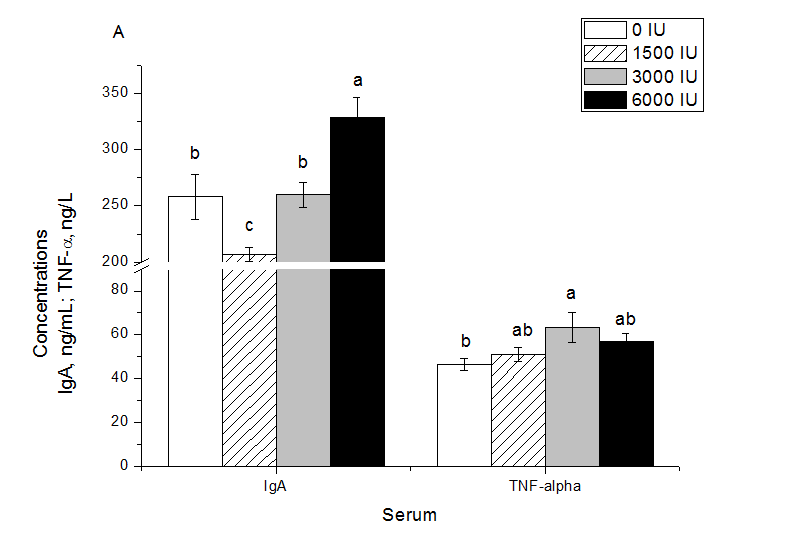


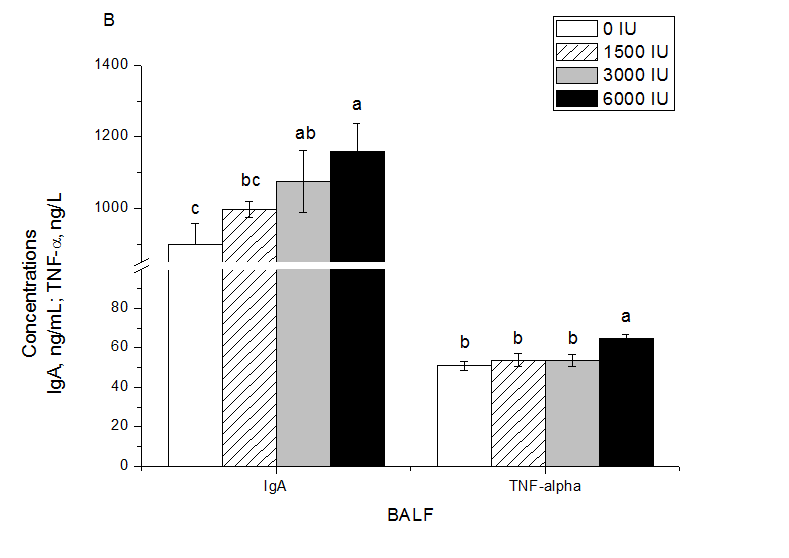


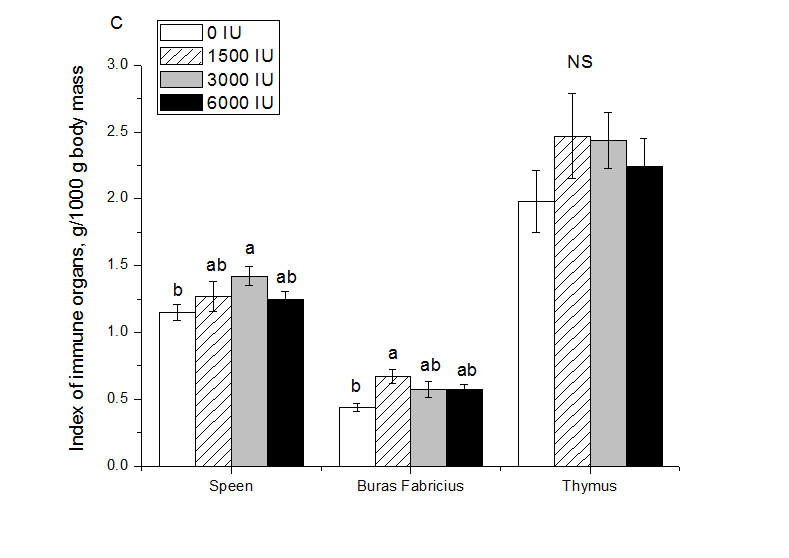

Supplement: S1 Fig — (DOC) [file pone.0139131.s002.doc]
